# PTPRG and PTPRC modulate nilotinib response in chronic myeloid leukemia cells

**DOI:** 10.18632/oncotarget.24253

**Published:** 2018-01-15

**Authors:** Julia Drube, Thomas Ernst, Markus Pfirrmann, Benadict Vincent Albert, Sebastian Drube, Daniela Reich, Anne Kresinsky, Kathrin Halfter, Claudio Sorio, Christian Fabisch, Andreas Hochhaus, Frank-D. Böhmer

**Affiliations:** ^1^ Institut für Molekulare Zellbiologie, CMB, Universitätsklinikum Jena, Jena, Germany; ^2^ Abteilung Hämatologie und Internistische Onkologie, Klinik für Innere Medizin II, Universitätsklinikum Jena, Jena, Germany; ^3^ Institut für Medizinische Informationsverarbeitung, Biometrie und Epidemiologie (IBE), Ludwig-Maximilians Universität, Munich, Germany; ^4^ Institut für Immunologie, Universitätsklinikum Jena, Jena, Germany; ^5^ Department of Medicine, University of Verona, Verona, Italy

**Keywords:** chronic myeloid leukemia, protein-tyrosine phosphatases, PTPRC, CD45, PTPRG

## Abstract

The introduction of second-generation tyrosine kinase inhibitors (TKIs) targeting the protein-tyrosine kinase (PTK) BCR-ABL1 has improved treatment response in chronic myeloid leukemia (CML). However, in some patients response still remains suboptimal. Protein-tyrosine phosphatases (PTPs) are natural counter-actors of PTK activity and can affect TKI sensitivity, but the impact of PTPs on treatment response to second-generation TKIs is unknown. We assessed the mRNA expression level of 38 PTPs in 66 newly diagnosed CML patients and analyzed the potential relation with treatment outcome after 9 months of nilotinib medication. A significantly positive association with response was observed for higher PTPN13, PTPRA, PTPRC (also known as CD45), PTPRG, and PTPRM expression. Selected PTPs were then subjected to a functional analysis in CML cell line models using PTP gene knockout by CRISPR/Cas9 technology or PTP overexpression. These analyses revealed PTPRG positively and PTPRC negatively modulating nilotinib response. Consistently, PTPRG negatively and PTPRC positively affected BCR-ABL1 dependent transformation. We identified BCR-ABL1 signaling events, which were affected by modulating PTP levels or nilotinib treatment in the same direction. In conclusion, the PTP status of CML cells is important for the response to second generation TKIs and may help in optimizing therapeutic strategies.

## INTRODUCTION

Chronic myeloid leukemia (CML) is caused by the reciprocal chromosomal translocation t(9;22) that results in the gene fusion of BCR (Breakpoint Cluster Region) and ABL1 (Abelson Murine Leukemia Viral Oncogene Homolog 1). The resulting gene product is the constitutively active protein-tyrosine kinase (PTK) BCR-ABL1. This oncogenic kinase leads to activation of mitogenic signal transduction pathways, resulting in the uncontrolled proliferation of myeloid cells and the development of chronic myeloid leukemia (CML) [1].

CML patients can be successfully treated with tyrosine kinase inhibitors (TKIs) targeting BCR-ABL1. Introduction of imatinib as a first targeted therapy for CML led to a major improvement in remission and overall survival rates [2]. The second generation BCR-ABL1 TKIs nilotinib and dasatinib are more potent, many patients reach a sustained deep molecular response (BCR-ABL1^IS^ ≤ 0.01 %, MR^4^ or better) [3]. Also, many patients with acquired point mutations within the BCR-ABL1 kinase domain, which cause imatinib-resistance, were successfully treated with these compounds [4]. In addition, a functional cure of a subset of patients in terms of treatment-free remission (TFR) can be achieved [5–7]. Current clinical trials, such as the German TIGER study, aim at improving the treatment regimes, allowing a higher rate of patients with deep molecular response to discontinue TKI treatment and stay in TFR. Despite the great improvements in treatment, some patients still do not reach deep molecular responses or require a prolonged time to do so. Others suffer from disease progression during treatment, independently from acquisition of resistance-causing mutations in BCR-ABL1 [1, 8]. Understanding the causes of these differences in response may improve treatment strategies.

Protein-tyrosine phosphatases (PTPs) are counter-actors of PTK signaling and can have either negative or positive regulatory functions in cancer-related signaling pathways [9, 10]. It is therefore plausible that the specific PTP status of cancer cells can have effects on therapy responses, especially for treatments with TKIs. Therefore, it has been previously considered that the expression levels of specific PTPs may modify the response to BCR-ABL1 inhibitors. For example, for imatinib responses, promoting effects of PTPN1 (PTP1B) [11], PTPN6 (SHP-1) [12], and PTPN2 (TC-PTP) [13, 14] were reported. However, for PTPN2 and PTPN6, findings were contradictory [15, 16]. Also, higher PTPN22 (Lyp) expression was linked to imatinib resistance in a genome-wide association study [17].

There is hitherto no comprehensive study addressing the role of PTPs for responses to second generation TKIs. We therefore addressed this issue in the current study and analyzed 38 PTPs in a cohort of 66 newly diagnosed CML patients treated with nilotinib (TIGER study). We investigated the influence of the PTP expression levels (before intended start of treatment) on response (reflected by BCR-ABL1^IS^) after 9 months of nilotinib treatment. For selected PTPs, we further studied the functional relevance in cell line models and identified PTPRG and PTPRC as modulators of intrinsic nilotinib sensitivity of BCR-ABL1 expressing cells.

## RESULTS

### PTP mRNA expression at start of treatment is associated with molecular response to nilotinib

**Figure 1:**
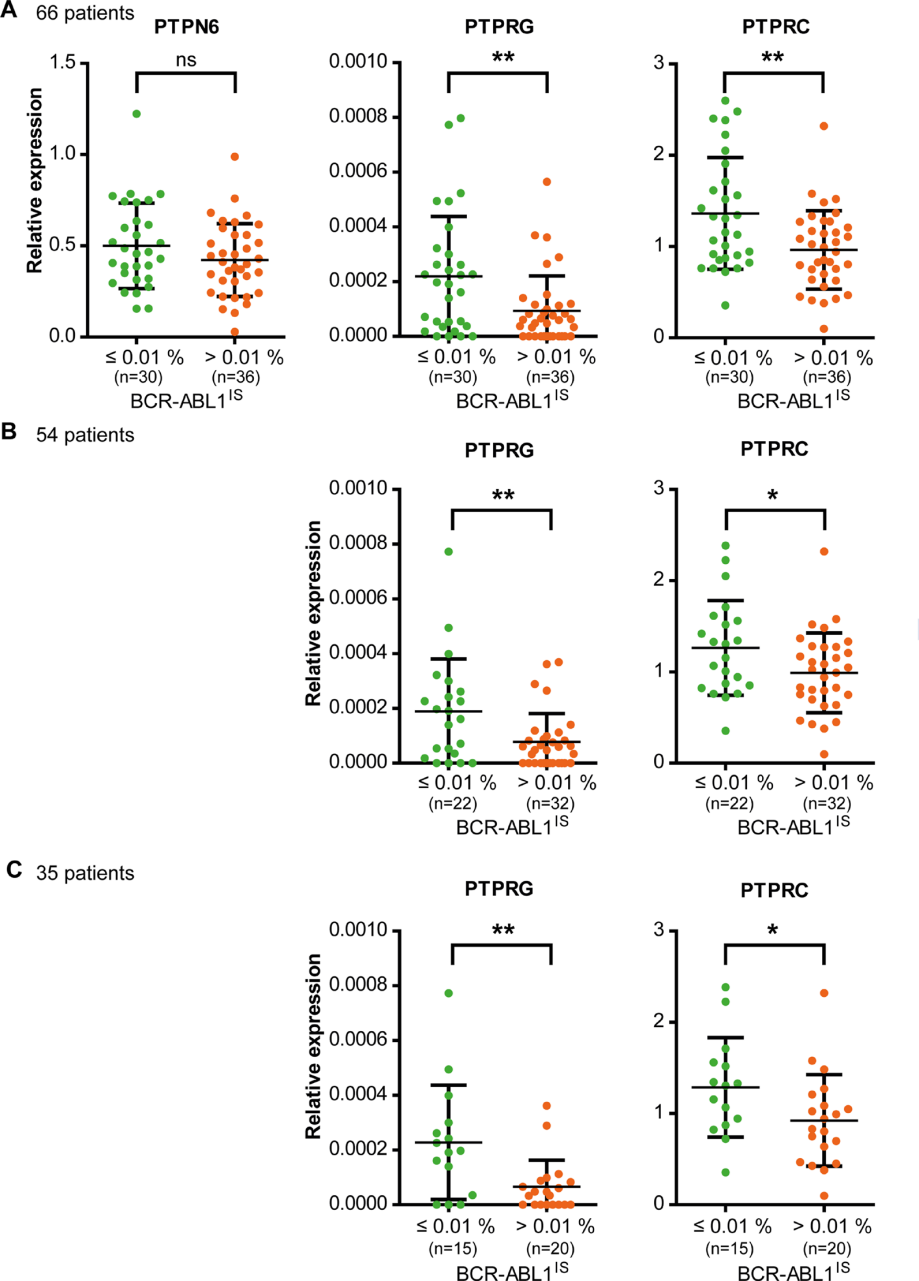
Expression levels of PTPRG and PTPRC, but not of PTPN6 at the beginning of nilotinib study treatment are associated with response after 9 months of treatment The RNA of total, peripheral blood leukocytes of CML patients in chronic phase was isolated and mRNA expression levels of 38 PTPs (Table S1) were analyzed. *GUSB* and *B2M* were used as control genes. (**A**) 66 patients were grouped according to their individual BCR-ABL1^IS^ (MR^4^ yes or no) after 9 months of nilotinib treatment. The mRNA levels for three selected PTPs in these patient categories are shown. Since the TIGER study protocol allowed inclusion of patients TKI pretreated for up to six weeks, and up to 6 months with hydroxyurea, we reassessed the actual treatment schedule of all 66 patients after unblinding. We defined two sub-cohorts of patients: (**B**) in one cohort we excluded 12 of 66 patients that had been TKI pretreated, or discontinued nilotinib within the first 9 months of treatment (*n* = 54). (**C**) In the second cohort, we additionally excluded all patients pretreated with hydroxyurea for more than 2 days, and patients that reduced nilotinib dose for more than 3 weeks (*n* = 35). Each point represents the relative PTP expression value of one patient, bars show mean values +/− standard deviation (SD). Differences in PTP mRNA expression between the patient groups were statistically tested. ns (not significant) if *p* > 0.05; ^*^ if *p* < 0.05; ^**^ if *p* < 0.01. P values were calculated using the likelihood ratio test. For details and confidence limits see Table 2.

To assess a potential influence of PTP expression on nilotinib response, 38 PTPs (Supplementary Table 1) were considered, which were previously shown to be expressed in hematopoietic cells including myeloid leukemia [18, 19], or have previously been shown to be dysregulated in cancer cells [9, 20, 21]. Blood samples of 66 patients (Table 1) were analyzed before start of treatment according to study protocol. PTP mRNA expression levels spanned a wide range, both among PTP genes and among individual patients (Supplementary Figure 1). The association of the PTP mRNA levels with response after 9 months of treatment was assessed using univariate logistic regression analyses. At this time, about equal numbers of patients had reached an MR^4^ (*BCR-ABL1*^IS^ ≤ 0.01 %; *n* = 30) or not (*BCR-ABL1*^IS^ > 0.01 %; *n* = 36). Higher mRNA levels for *PTPRG* (*RPTPγ*) and *PTPRC* (*CD45*) (Figure 1, Table 2) as well as for *PTPN13* (*FAP1, PTP-BAS*), *PTPRA* (*RPTPα*), and *PTPRM* (*RPTPμ*) (Table 2) significantly supported MR^4^ probabilities after 9 months of treatment. Notably, no significant associations were found for PTPN1 (PTP1B), PTPN2 (TC-PTP), PTPN6 (SHP-1; Figure 1), or PTPN22 (Lyp), PTPs whose expression had previously been reported to positively associate with imatinib response [11–14, 17].

**Table 1:** Patient characteristics

Total number of patients	66
Age	Median 50 [19 – 72] IQR: [36 – 57]
Gender	female *n* ***=*** 18 (27.3 %)
EUTOS-Score	low risk *n* ***=*** 56 (84.8 %)
WBC x10^9^/L	Median 48.45 [3.5 – 555] IQR: [18.95 – 160.55]^*^)
Platelets x10^9^/L	Median 405.5 [93 – 3255] IQR: [256 – 670.5]^*^)
Hb g/dL	Median 12.25 [8.3 – 16.2] IQR: [10.3 – 14]^**^)

^*^) 2 values missing; ^**^) 4 values missing.

**Table 2:** Odds ratios in univariate logistic regression on the probability to be in MR^4^ nine months after start of nilotinib therapy

Patient cohorts, number of patients (*n*)	PTP	Odds ratio	Lower 95% confidence limit for odds ratio	Upper 95% confidence limit for odds ratio	*P* value likelihood ratio test
*n* ***=*** 66	*PTPN13*	1.045	1.009	1.092	0.0122
	*PTPRA*	1.275	1.073	1.561	0.0047
	***PTPRC***	**1.015**	**1.005**	**1.028**	**0.0024**
	***PTPRG***	**1.046**	**1.013**	**1.088**	**0.0039**
	*PTPRM*	1.019	1.004	1.038	0.0154
*n* ***=*** 54	*PTPN13*	1.040	0.995	1.093	0.0834
	*PTPRA*	1.287	1.056	1.628	0.0112
	***PTPRC***	**1.013**	**1.001**	**1.027**	**0.0389**
	***PTPRG***	**1.059**	**1.015**	**1.116**	**0.0064**
	*PTPRM*	1.012	0.995	1.031	0.1748
*n* ***=*** 35	*PTPN13*	1.039	0.990	1.103	0.1242
	*PTPRA*	1.344	1.060	1.826	0.0123
	***PTPRC***	**1.014**	**1.000**	**1.031**	**0.0430**
	***PTPRG***	**1.087**	**1.026**	**1.175**	**0.0023**
	*PTPRM*	1.032	1.005	1.069	0.0168

PTPs analyzed in cell based assays are highlighted. For cohort details see legend Figure 1.

Potential correlations of PTP mRNA expression levels with clinical parameters were also assessed but no substantial associations were found (data not shown).

Since the TIGER study protocol allowed inclusion of patients that had been pre- treated with nilotinib or imatinib for up to six weeks, and with hydroxyurea for up to six months, we also assessed patient subgroups excluding pretreatments and other deviations from the treatment schedule, which might have been important in the context of our study. We defined two subgroups comprising 54/66 or 35/66 patients, with only minor or no pretreatment, or virtually no pretreatment, respectively (for details see legend Figure 1). We reanalyzed the two cohorts for the 5 PTPs initially found to be associated with nilotinib treatment response. The significance was maintained for PTPRA, PTPRC, and PTPRG, but was lost for PTPRM in the *n* = 54 subgroup and for PTPN13 in both subgroups (Figure 1, Table 2).

### PTPRG and PTPRC, but not PTPN6 modulate nilotinib response in cell lines

Among the five PTPs associated with response, we focused our subsequent analyses on PTPRG and PTPRC. Apart from reasons of technical feasibility, this decision was based on the following considerations: PTPRG had earlier been shown to negatively regulate BCR-ABL1 mediated transformation [22]. PTPRC is highly expressed, and an influence of this PTP on signal transduction in leukemia was previously reported [23–26]. In addition, PTPN6 was further investigated although no influence was observed in our analysis. However, it is abundantly expressed in patient samples and previous data suggested a role for TKI response [12].

The expression levels of the selected three PTPs were manipulated in CML cell lines, and the effect on TKI sensitivity was analyzed.

**Figure 2:**
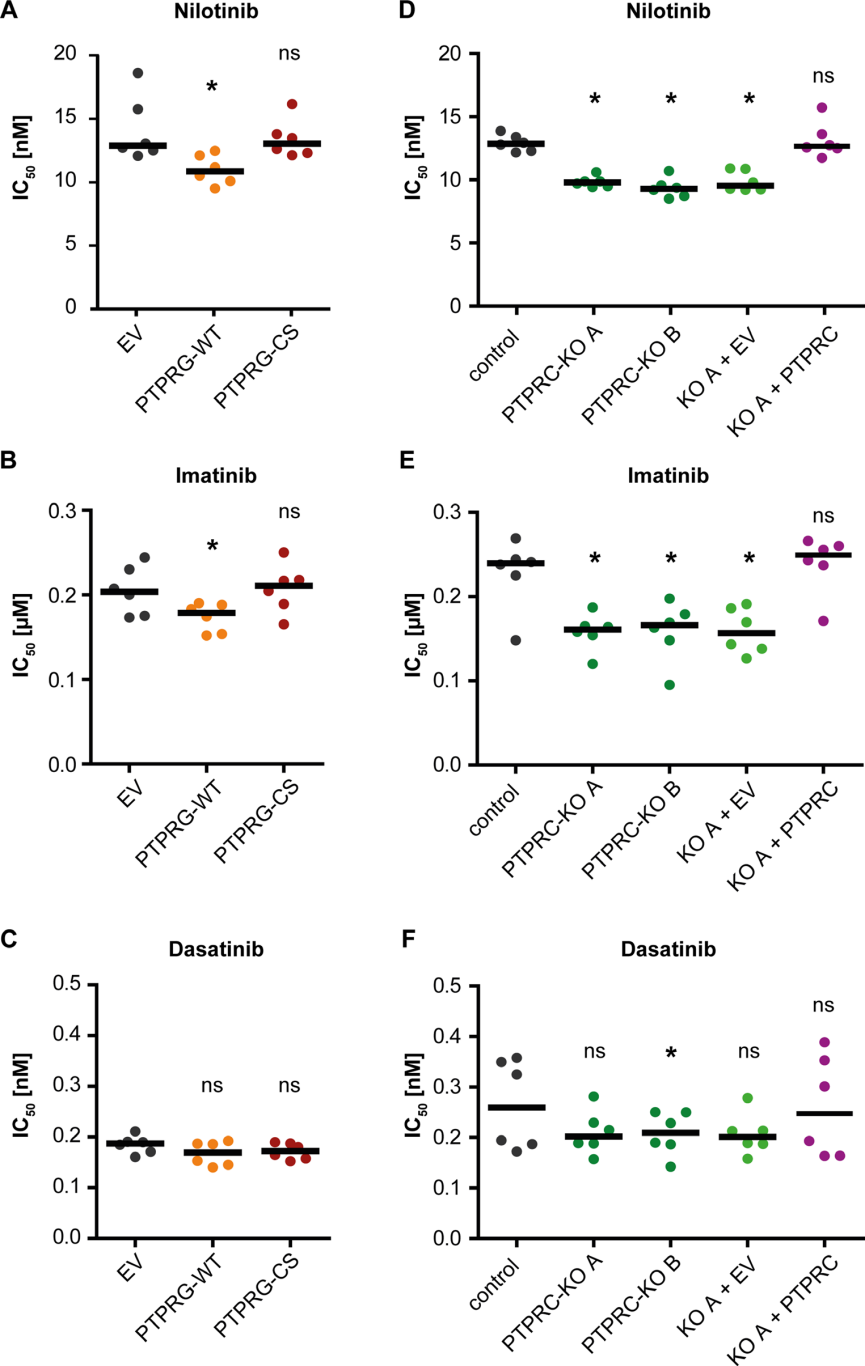
PTPRG and PTPRC modulate nilotinib and imatinib response in cell lines IC_50_ analyses for nilotinib (A, D), imatinib (B, E), and dasatinib (C, F) were carried out with K562 cells stably expressing wild type PTPRG (WT) or catalytically inactive PTPRG-C1060S mutant (CS) (**A**, **B**, **C**) or with two individual clones (A and B) of K562 cells subjected to CRISPR/Cas9 mediated knockout of *PTPRC* gene and corresponding CRISPR/Cas9 control cells. Additionally, PTPRC-KO clone A was used to re-express PTPRC by lentiviral transduction (KO A + PTPRC; control: KO A + EV (empty vector)) (**D**, **E**, **F**). PTPRG and PTPRC protein levels of engineered cells are shown in Figures 5 and 6 respectively. Each dot represents the IC_50_ value determined in one independent experiment with quadruplicate technical replicas, black bars show the median of *n* = 6 independent experiments. ns (not significant) if *p* > 0.05; ^*^ if *p* < 0.05. Comparison with the respective EV or control was performed with the Wilcoxon matched pairs test.

PTPRG levels are quite low in primary CML cells (Figure 1, Supplementary Figure 1) and K562 cells [22], a cell line model widely used for analyzing BCR-ABL1 inhibitors. We therefore overexpressed wild type PTPRG (WT) or the catalytically inactive PTPRG-C1060S (CS) mutant in K562 cells. PTPRG protein expression in the resulting cell pools is shown in Figure 5. We then assessed the effects on TKI responsiveness. Cell pools were subjected to treatment with dose-ranges of nilotinib, imatinib, and dasatinib and effects were measured using an IC_50_ assay (Supplementary Figure 2). PTPRG-WT overexpression caused an improved effect of nilotinib (Figure 2A, Supplementary Figure 2B) and imatinib (Figure 2B) indicated by a lowered IC_50_, whereas TKI effects on the PTPRG-CS mutant-expressing cells were not significantly different from the controls. For dasatinib, we did not observe significant differences for the different cell pools (Figure 2C).

**Figure 3:**
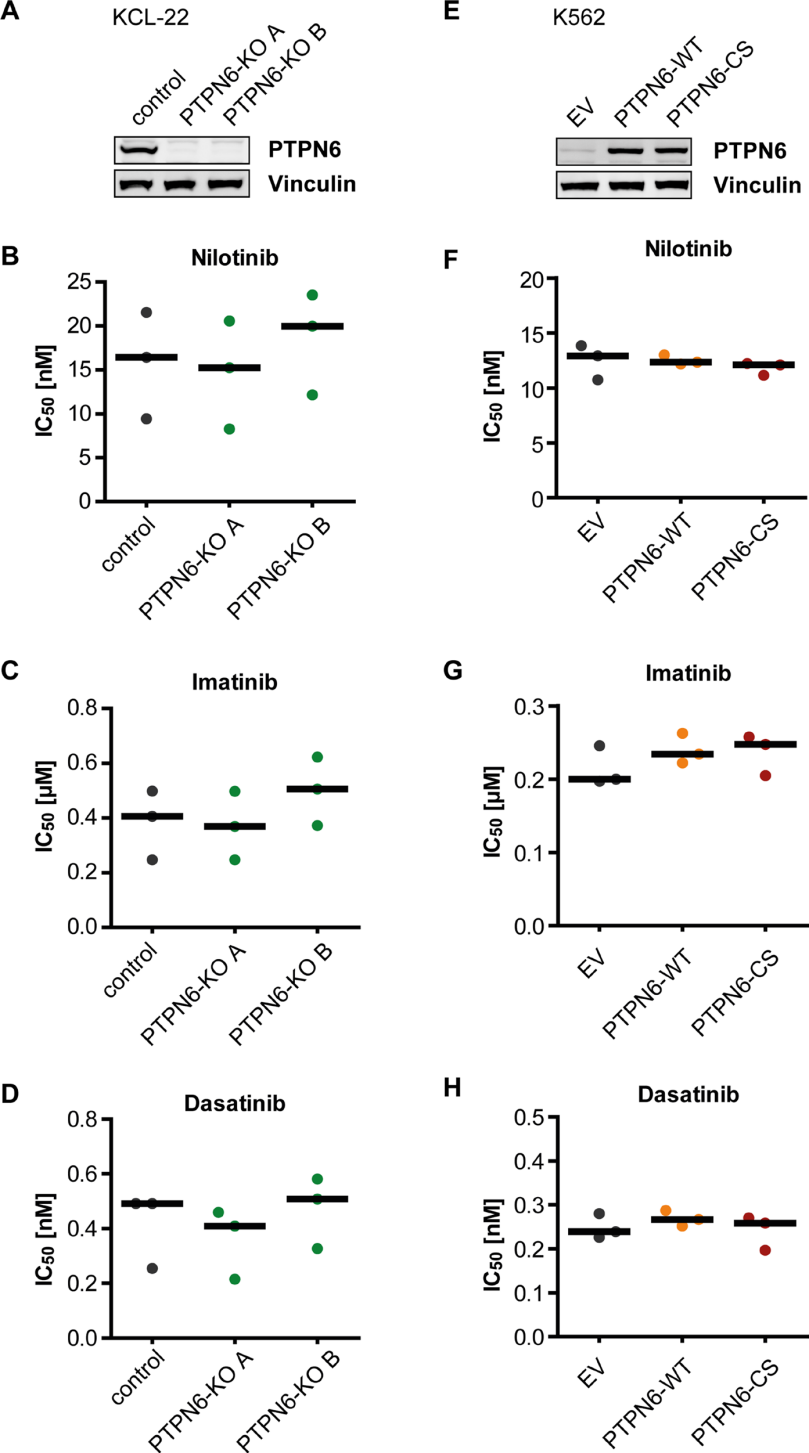
PTPN6 expression and response of CML cells to TKI KCL-22 Cells were subjected to CRISPR/Cas9 mediated knockout of the *PTPN6* gene. Two individual single cell clones were analyzed for PTPN6 expression using western blot (**A**). K562 cells with low endogenous PTPN6 expression were stably transduced with either empty vector (EV), wild type PTPN6 (WT), or the catalytically inactive PTPN6-C453S (CS) mutant. Protein expression was analyzed by western blot (**E**). The engineered cells were analyzed for IC_50_ of nilotinib (**B**, **F**), imatinib (**C**, **G**), and dasatinib (**D**, **H**). Each dot represents the IC_50_ value determined in one independent experiment with quadruplicate technical replicas, black bars show the median of *n* = 3 independent experiments.

**Figure 4:**
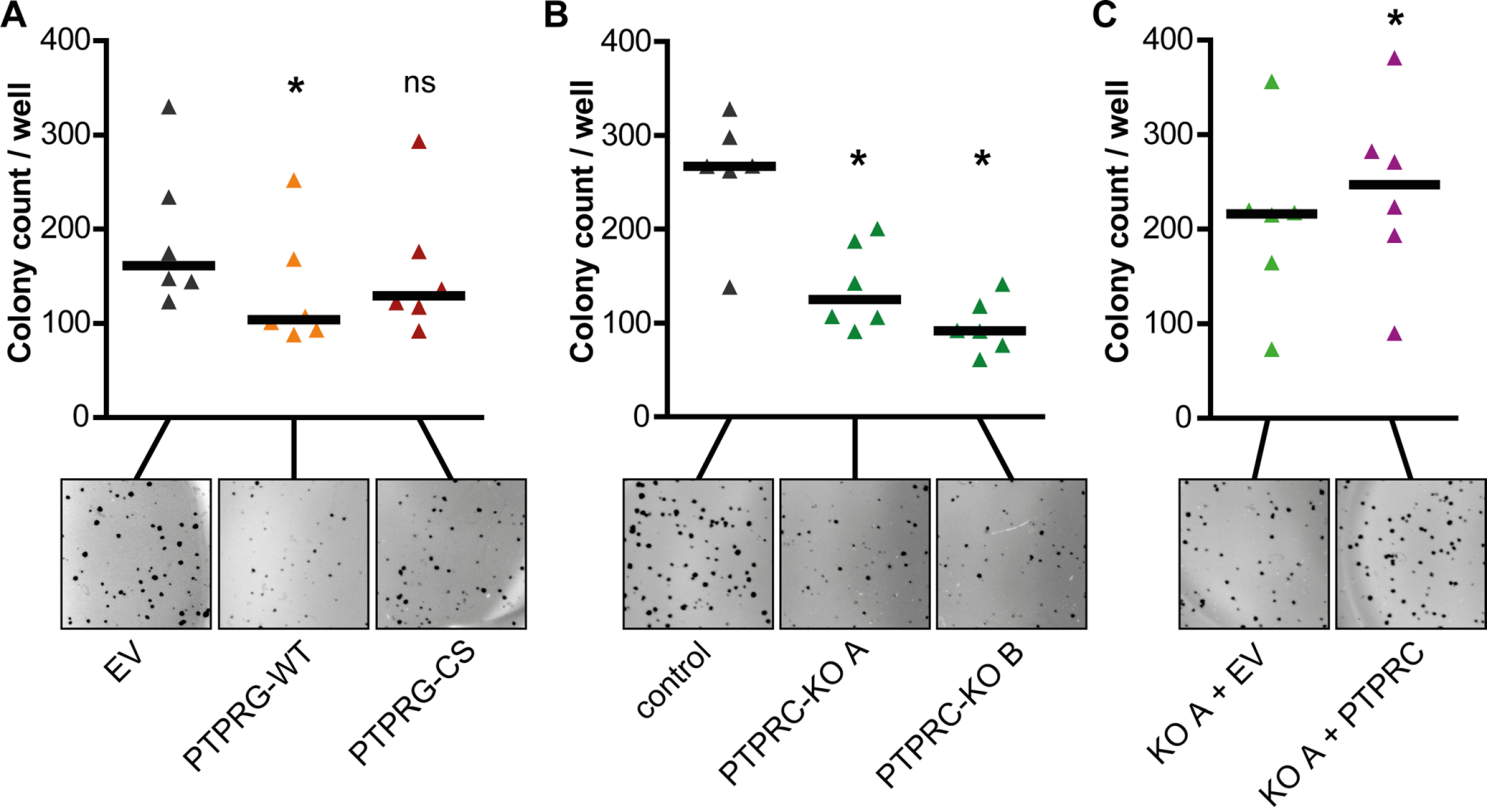
PTPRG attenuates and PTPRC promotes BCR-ABL1 dependent cell transformation K562 overexpressing PTPRG-WT or PTPRG-CS (**A**), *PTPRC* knockout clones A and B (**B**), or PTPRC re-expressing cells (KO A + PTPRC) (**C**) were analyzed for colony formation in methylcellulose. Representative images of colonies are shown. Each triangle represents colony count per well (mean of duplicates of an individual experiment). Black bars represent the median of *n* = 6 independent experiments. ns (not significant) if *p* > 0.05; ^*^ if *p* < 0.05. Comparison with the respective control was performed with the Wilcoxon matched pairs test.

**Figure 5:**
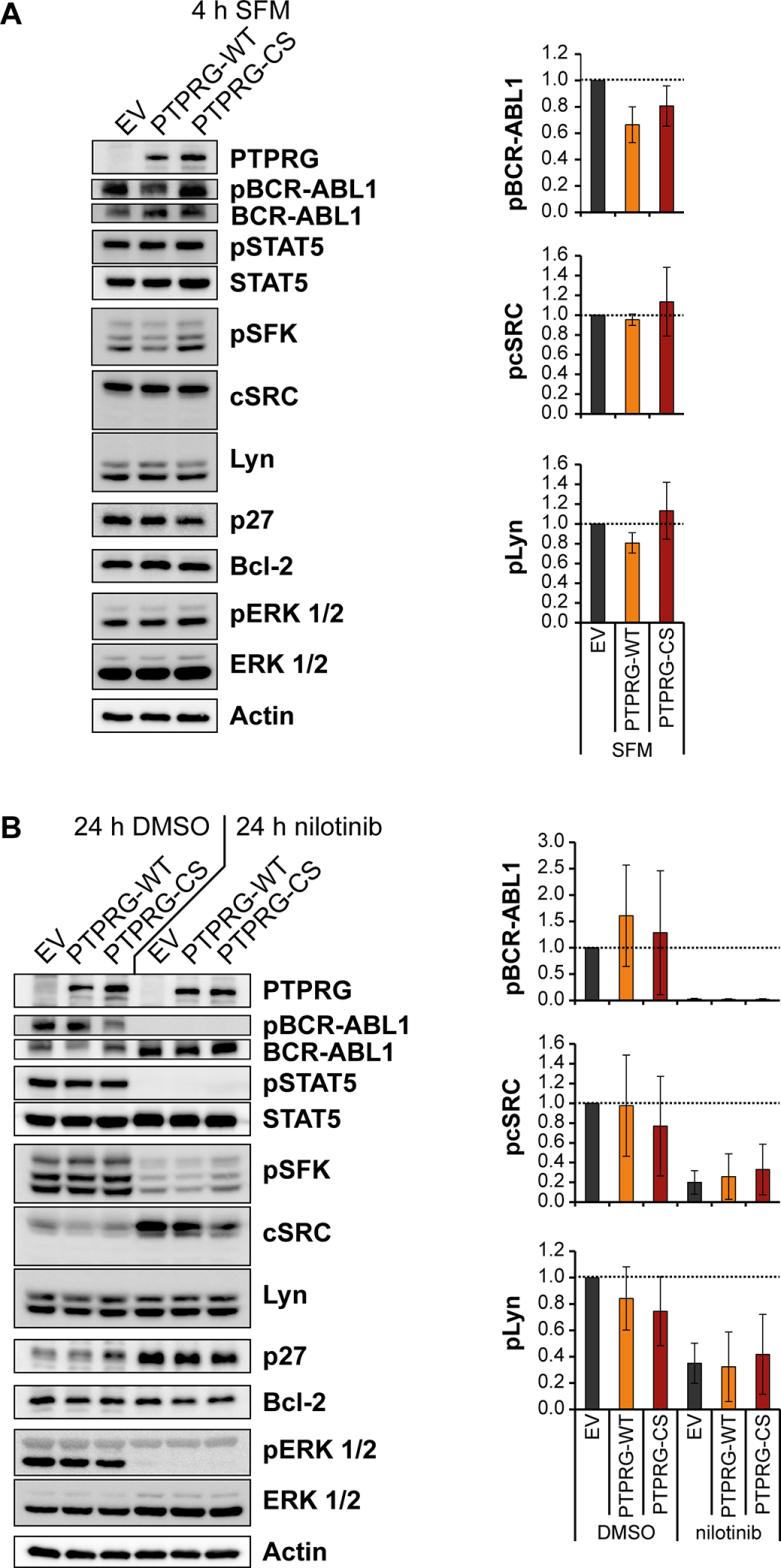
Nilotinib treatment and PTPRG overexpression affect BCR-ABL1 signal transduction in the same direction K562 cells overexpressing PTPRG-WT or PTPRG-CS were starved from serum for 4h (SFM) (**A**), or treated with nilotinib (1 μM, 24 h) or DMSO in presence of 10 % FCS (**B**). Cell lysates were prepared and subjected to SDS-PAGE and immunoblotting with the indicated antibodies. Total protein was detected after stripping off the respective phospho-specific antibodies. Representative blots of *n* = 3 independent experiments are shown (left). Quantification of blots from *n* = 3 independent experiments is shown on the right: the phospho-signal was divided by the respective total protein signal. The signals for PTPRG-WT and PTPRG-CS are reported relative to the control (EV) cell signals of each experiment. Shown is the mean relative intensity of *n* = 3 independent experiments +/− standard deviation (SD). Quantifications of pSTAT5, p27, Bcl-2, and pERK 1/2 are depicted in Supplementary Figure 4.

**Figure 6:**
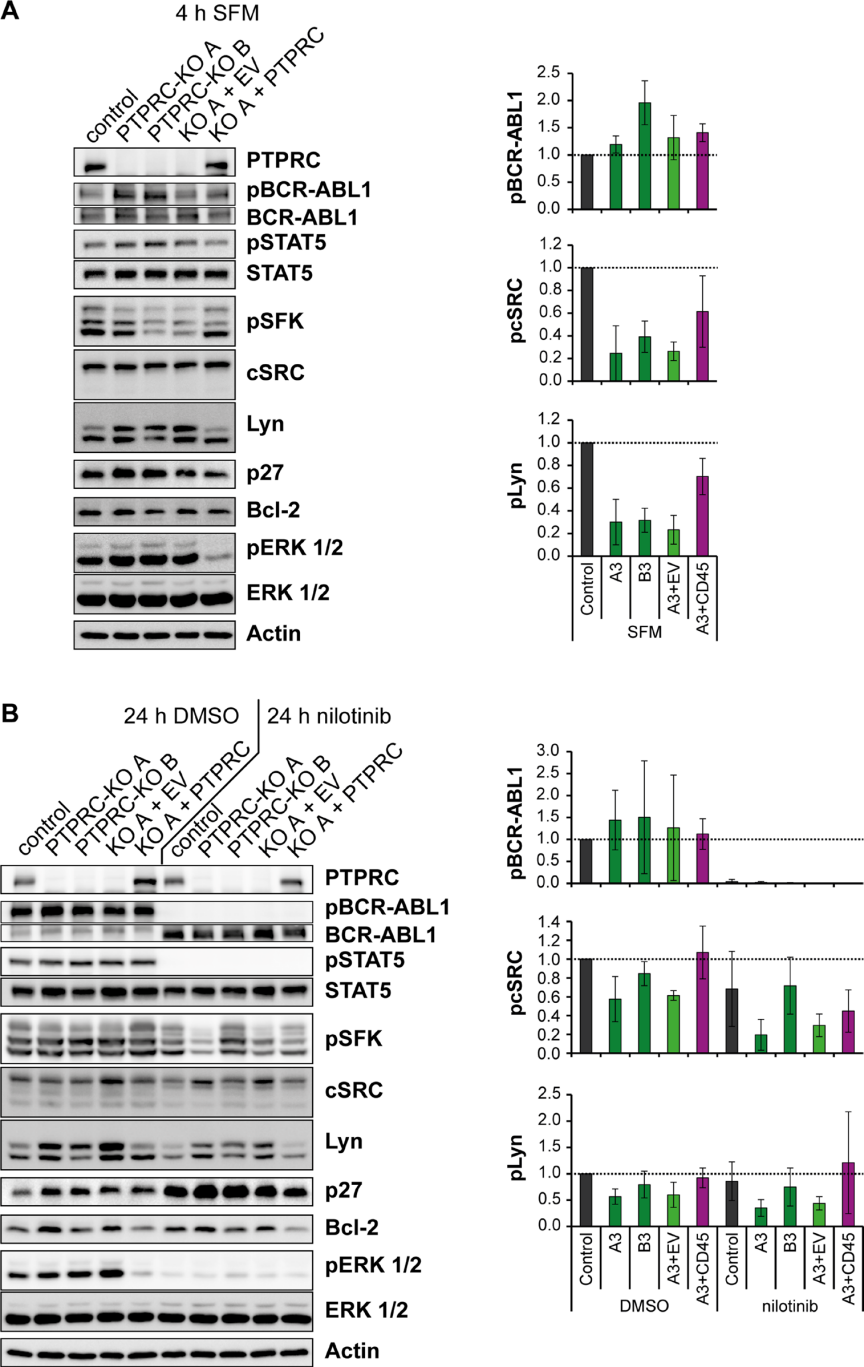
Nilotinib treatment and PTPRC deficiency affect aspects of BCR-ABL1 signal transduction in the same direction K562 cells with *PTPRC* knockout and respective PTPRC re-expressing cells were starved from serum for 4h (SFM) (**A**), or treated with nilotinib (1 μM, 24 h) or DMSO in presence of 10 % FCS (**B**). Cell lysates were prepared and subjected to SDS-PAGE and immunoblotting with the indicated antibodies. Total protein was detected after stripping off the respective phospho-specific antibodies. Representative blots of *n* = 3 independent experiments are shown (left). Quantification of blots of *n* = 3 independent experiments is shown on the right: the phospho-signal was divided by the respective total protein signal. The signals of engineered PTPRC KO and re-expressing cells are reported relative to the control cell signals of each experiment. Shown is the mean relative intensity of *n* = 3 independent experiments +/− standard deviation (SD). Quantifications of pSTAT5, p27, Bcl-2, and pERK 1/2 are depicted in Supplementary Figure 5.

PTPRC was highly expressed in primary CML cells (Figure 1, Supplementary Figure 1), and CML cell lines (data not shown). Therefore we chose to perform a CRISPR/Cas9-mediated knockout in K562 cells to assess its impact on TKI sensitivity. Control cells were transduced with a CRISPR/Cas9 construct lacking a guide sequence. Analyses of two independent *PTPRC* knockout clones and a cell pool derived from one knockout clone by rescue with exogenous human PTPRC were performed. Expression controls for PTPRC protein for all analyzed cells are shown in Figure 6. The *PTPRC* knockout caused an improved response to nilotinib and imatinib indicated by a lowered IC_50_, which was reverted by exogenous PTPRC expression (Figure 2D, 2E, Supplementary Figure 2C). The differences for dasatinib were not significant for most comparisons (Figure 2F).

Since K562 cells have very low endogenous PTPN6 expression, we employed KCL-22 cells, which express PTPN6 prominently, to assess the effect of PTPN6 on TKI response. Knockdowns with two different shRNAs (Supplementary Figure 3A) did not reveal consistent changes in TKI responses, but for one shRNA suggested a potentially higher sensitivity for nilotinib and imatinib (Supplementary Figure 3B, 3C). We then used the CRISPR/Cas9 technology for generating KCL-22-*PTPN6* knockout cell clones (Figure 3A), and also overexpressed either wild type PTPN6 (WT) or the catalytically inactive PTPN6-C453S (CS) mutant in K562 cells (Figure 3E). Neither of these manipulations in PTPN6 levels appeared potentially associated with alterations in IC_50_ for any of the three tested TKIs (Figure 3B–3D; 3F–3H).

### PTPRG attenuates and PTPRC promotes BCR-ABL1 dependent cell transformation

We analyzed the effects of PTPRG and PTPRC on BCR-ABL1 dependent cell transformation by performing colony formation assays in methylcellulose.

Overexpression of PTPRG-WT in K562 cells moderately attenuated colony formation, whereas the expression of the catalytically inactive PTPRG-CS had no significant effect (Figure 4A).

For PTPRC, the CRISPR/Cas9 mediated *PTPRC* knockout was associated with a reduced colony growth of engineered cells in methylcellulose (Figure 4B). Analysis of cells with re-introduction of exogenous PTPRC was hampered by enhanced colony formation already in cells transduced with the empty lentiviral control vector (EV). Still, the extent of colony formation was even further enhanced with exogenous PTPRC (Figure 4C).

These findings suggest that BCR-ABL1 dependent cell transformation is attenuated by PTPRG and promoted by PTPRC.

### Nilotinib treatment, PTPRG overexpression, and PTPRC deficiency affect BCR-ABL1 signal transduction in the same direction

We also assessed effects of altered PTP levels on signal transduction in the engineered cells, with or without nilotinib treatment. We first screened a range of signaling molecules and cell cycle regulators known to be influenced by BCR-ABL1 activity. Based on the initial findings, we chose to further analyze BCR-ABL1 autophosphorylation, the activation of Src-family tyrosine kinases (SFK), signal transducer and activator of transcription 5 (STAT5), and extracellular signal regulated kinase 1 and 2 (ERK 1/2), as well as the expression levels of p27, a cell-cycle inhibitor, and Bcl-2, an inhibitor of apoptosis. The analysis was done in absence of serum for 4 h to eliminate potential effects of serum-derived growth factors that might cover up signal changes, or in complete culture medium for 24 h, without and with nilotinib treatment, similar to the conditions for IC_50_ determination.

Overexpression of PTPRG-WT, but not of PTPRG-CS had an inhibitory effect on BCR-ABL1 autophosphorylation, visible in the absence of serum, and potentially also on the phosphorylation of the SFK Lyn (Figure 5A). Note that the exact identity of the detected active SFK in the pSFK blot is not known. Blots were re-probed with antibodies for the two likely candidates by size, cSRC and Lyn, revealing presence and equal loading for both, and allowing quantification. In presence of serum, PTPRG-WT moderately diminished the phosphorylation of ERK 1/2 (Figure 5B, Supplementary Figure 4B). No effect of PTPRG was seen for STAT5 activation (Figure 5, Supplementary Figure 4). Nilotinib completely eliminated activation of most of these signaling mediators, except that inhibition of SFK was only partial (Figure 5B, Supplementary Figure 4B).

PTPRC loss by CRISPR/Cas9 knockout caused moderately increased BCR-ABL1 phosphorylation, a robust ERK1/2 activation, and an increase of p27 expression, with the latter only seen in presence of serum (Figure 6, Supplementary Figure 5). Interestingly, PTPRC loss resulted in a pronounced decrease of SFK activity, seen prominently upon analysis in absence of serum (Figure 6A) and moderately also in presence of serum (Figure 6B). Notably, all the PTPRC knockout effects were rescued by re-expression of exogenous PTPRC. These observations suggest that PTPRC can modulate BCR-ABL1 signaling both negatively and positively, by differentially affecting specific signaling outputs. Reduced SFK activity in absence of PTPRC, and potentially also elevated p27 may be critical for attenuated colony formation and improved response to nilotinib.

## DISCUSSION

This study provides a first comprehensive analysis of the potential association of PTP expression with response to the second-generation TKI nilotinib in CML patients and cell lines. We identified several PTPs whose expression was associated with a more efficient response to this TKI, including PTPRG and PTPRC. Using genetically engineered cells, PTPRG and PTPRC were shown to causally affect the nilotinib sensitivity at cellular level. PTPRG overexpression and *PTPRC* knockout using CRISPR/Cas9 technology enhanced the nilotinib response.

The expression levels of PTPN13, PTPRA, and PTPRM were also observed to significantly support MR^4^ probabilities after 9 months in our 66 patient cohort although these associations were partially lost for PTPN13 and PTPRM with more stringently selected patient populations. These associations further indicate the relevance of PTP gene expression for TKI responses in CML, and the potential functions of these PTPs at cellular level need to be analyzed in future studies.

Several PTPs previously reported to modulate the response to the first-generation inhibitor imatinib, including PTPN1 [11], PTPN2 [13, 14], and PTPN22 [17] were not associated with response in our study. It is possible that the more efficient inhibition of BCR-ABL1-mediated transformation by nilotinib may override PTP-mediated regulation in these cases. We could also not confirm a previously reported role of PTPN6 [12]. Notably, in our study, potential PTPN6 effects were directly assessed in engineered cell lines (overexpression, knockdown, and knockout of PTPN6) and an importance of this PTP was not detectable for any of the tested TKI. These findings are in line with a recent report on imatinib sensitivity from another laboratory [16].

Consistent with the effect on nilotinib and imatinib response, PTPRG overexpression and *PTPRC* knockout both attenuated BCR-ABL1 mediated transformation as detectable in colony formation assays. Similar effects of PTPRG were reported earlier from one of our laboratories [22]. Inhibition of transformation correlated with the inhibition of SFK activation, an aspect of transforming BCR-ABL1 signaling, seen in a pronounced manner for the *PTPRC* knockouts, and with a moderate elevation of the cell-cycle inhibitor p27, in case of PTPRC deficiency. Nilotinib affected these molecules in the same direction. We therefore suggest that the effects on the related signaling pathways form the basis for the enhanced nilotinib response in PTPRG overexpressing or PTPRC deficient cells. Interestingly, modulation of PTPRG or PTPRC levels in cell lines had considerably less effect on responses to dasatinib than on those to nilotinib or imatinib. It is tempting to speculate that concurrent inhibition of BCR-ABL1 and SFKs by dasatinib may eliminate the differences in BCR-ABL1 signaling imposed by these PTPs.

PTPRG is expressed at low levels in CML cells, but the PTPRG expression is not negatively regulated by BCR-ABL1 (data not shown) and low PTPRG levels in CML cells may be the result of a long-term selection process, possibly based on a small growth advantage of cells with lower PTPRG levels caused by *PTPRG* promoter methylation [22, 27]. Consistent with our cell line studies, higher PTPRG mRNA levels in the patient study were associated with an excellent nilotinib response. Therefore, the potential usefulness of PTPRG for prediction of response should be further investigated.

PTPRC was identified in our study as a previously unrecognized positive mediator of BCR-ABL1 mediated cell transformation. Signaling of BCR-ABL1 was differentially affected by PTPRC. PTPRC is a *bona-fide* PTP for inhibitory phosphotyrosines on SFKs such as the SFK LCK56 in T-cells [28]. This is consistent with the notion that we found SFK activity diminished in PTPRC-deficient cells. However, BCR-ABL1 autophosphorylation and ERK1/2 activation appeared negatively regulated by PTPRC. The dual effects of PTPRC on BCR-ABL1 signaling are reminiscent of its role in T-cells, where it both positively and negatively modulates T-cell receptor signaling. Clearly, the positive regulation of BCR-ABL1 mediated transformation by PTPRC played a dominating role in our context. Surprisingly, the studies of causal effects of PTPRC in cell lines were contrasting with the direction of association of PTPRC mRNA levels in the patient study, in which higher PTPRC mRNA levels appeared related to a better response. PTPRC mRNA expression was not substantially associated with leukocyte counts (not shown). However, the analyzed total leukocytes comprise a range of different cell populations, among which PTPRC is differentially expressed [18]. The presence of cell populations with particular high PTPRC expression may indicate better responses, in contrast to the intrinsic role of this PTP for transformation and TKI-susceptibility at the cellular level as it was identified in the functional analysis. Clearly, the identity of PTPRC expressing cells, which correlate with good response, remains to be identified.

Improving the efficiency of TKI response in CML is still of interest to achieve deep molecular responses in shorter times and to eradicate CML cells more efficiently. Both are prerequisites for allowing discontinuation of TKI treatment with low probability of recurrence of the disease. The mean reductions of IC_50_ for nilotinib observed in PTPRG overexpressing or PTPRC knockout-cells were in the range of 20–30%. Given the side effects of the drug and the need of rapidly achieving an MR^4^, we believe that these quantitative differences are clinically meaningful. Activation of PTPs positively modulating response or inhibition of PTPs associated with less efficient response would be potentially beneficial in this respect. While PTPs themselves have emerged as difficult drug targets [29], the signaling pathways subject to PTP-mediated regulation may be informative for the identification of possible auxiliary therapeutic approaches. From our analysis of PTP-modulated signaling, inhibition of SFKs and elevation of p27 levels emerged as potential goals for related strategies. Potentially, PTP mRNA levels may help identifying patients with particularly good prognosis and enable treatment schedules with lesser side effects, e.g. by dose reduction. The potential suitability of PTPs as biomarkers will require, however, further studies in additional patient cohorts.

In summary, our study revealed that PTPs do modify TKI response in CML cells in the context of the second generation TKI nilotinib. PTPRG and PTPRC affect TKI sensitivity in opposite manner, consistent with their effects on BCR-ABL1 signal transduction and cell transformation.

## MATERIALS AND METHODS

### Patient characteristics and BCR-ABL1^IS^ determination

A total of 66 newly diagnosed CML patients in chronic phase (18 female, median age 50 years, range 19–72 years) were analyzed. Patients were treated within the German prospective multicenter phase 3 trial (TIGER trial; NCT01657604) receiving nilotinib (300 mg BID) alone (*n* = 31) or in combination with Peg-interferon α2b (30–50 μg/week) (*n* = 35) (Table 1). Further patient characteristics, including sub-cohorts, are summarized in the legend of Figure 1. All patients gave informed consent and the study was approved by the institutional ethics committees. Peripheral blood samples were collected at the time of inclusion in the study for evaluation of the underlying *BCR-ABL1* transcript. Total RNA was extracted after hypotonic red cell lysis from at least 20 ml of peripheral blood using the RNeasy Mini Kit (Qiagen, Hilden, Germany) or TRIzol reagent (Invitrogen, Carlsbad, CA, USA) according to the manufacturers’ instructions. Complementary DNA synthesis was performed using random hexamer primers and Moloney murine leukemia virus reverse transcriptase (Invitrogen) as described elsewhere [30]. *BCR-ABL1* and total *ABL1* transcripts were amplified using the LightCycler technology (Roche Diagnostics, Mannheim, Germany) and detected via specific hybridization probes as described previously [31, 32]. Two microliters of cDNA was used as the template for the quantitative real-time PCR reactions. *BCR-ABL1*^IS^ transcript levels were determined and reported according to the International Scale (IS) [33].

### RT-qPCR

Quantitative PCR primers (Table S1) for PTPs were designed using the NCBI primer-BLAST tool. Care was taken to pick up all transcript variants. *Beta-glucuronidase* (*GUSB*) and *Beta-2 microglobulin* (*B2M*) were used as control genes. The conditions for qPCR are described in the Supplementary Methods.

### Statistical analyses

To identify the prognostic influence of the candidate variables on achieving *BCR-ABL1*^IS^ ≤ 0.01 % (MR^4^) status at 9 months (yes or no), univariate logistic regression analyses were performed [34]. Significance was judged using the likelihood ratio test. Since the study has an exploratory character, multiplicity was not considered. For the two-sided *P* values, the unadjusted significance level was 0.05. All calculations were performed with the SAS software version 9.4 (SAS Institute, Cary, NC, USA).

For the *in vitro* experiments, the Wilcoxon matched pairs test was employed using GraphPad Prism 5. Note that comparison of 6 independent values is the minimum sample number for this test, which potentially meant a limitation to our study.

### Cells, cultivation, and cell treatments

K562 and KCL-22 cells were purchased from DSMZ (Braunschweig, Germany). Cells were cultured in RPMI1640 (Sigma-Aldrich, Deisenhofen, Germany, R8758) supplemented with 1 % penicillin/streptomycin (P/S; Sigma-Aldrich, P0781), and 10 % fetal calf serum (FCS).

### IC_50_ assay

10 000 cells were seeded in 125 μl growth medium containing either 0.2, 1, 5, 20, 100, 500, or 2 000 nM nilotinib, 0.005, 0.02, 0.1, 0.5, 2, 10, or 50 μM imatinib, or 0.01, 0.05, 0.2, 1, 5, 20, or 100 nM dasatinib, all with final DMSO concentration of 0.1 %. Selected experiments were carried out with final concentrations of 0.2, 1, 2.5, 5, 7.5, 10, 15, 20, 50, 100, 2 000 nM nilotinib. Each setting was seeded in quadruplicates. After incubation at 37°C for 72 h , 25 μl of CellTiter-Blue reagent (Promega, G8081) were added to each well, and after 2 hours of additional incubation, fluorescence (E_x_ 540 nm, E_m_ 610 nm) was measured using a TECAN Infinite 200 (Tecan, Crailsheim, Germany) plate reader. IC_50_ values were calculated using Sigma Plot 13.0.

### Colony formation assay

Methylcellulose stock solution (R&D Systems, Wiesbaden-Nordenstadt, Germany; HSC001) was diluted with IBM (Biochrom, FG0465) and supplemented with 10 % FCS and 1 % P/S, according to the instructions of the supplier. 750 K562 cells were seeded in 500 μl of the prepared methylcellulose mix per well in 24-well-plates in duplicates. After six days of cultivation at 37°C, 40 μl of a 4 mg/ml Iodonitrotetrazolium chloride (Sigma-Aldrich, I8377) solution was added dropwise onto the methylcellulose. The 24-well-plate with the stained colonies was scanned using a HP Scanjet G4050 scanner with 1 200 dpi resolution after additional incubation over night at 37°C. Colony counting was done using NIH ImageJ 1.47v software.

### Other methods and reagents

DNA constructs, cell engineering using viral transduction and CRISPR/Cas9 technology, antibodies, and signaling analyses are described in Supplementary Methods.

## SUPPLEMENTARY MATERIALS FIGURES AND TABLE


