# Supplementary material for: PTPRG and PTPRC modulate nilotinib response in chronic myeloid leukemia cells
Source: Oncotarget. 2018 Jan 15;9(10):9442–55. doi: 10.18632/oncotarget.24253 (PMC5823647; doi:10.18632/oncotarget.24253)
Supplement: Supplementary file 1 [file oncotarget-09-9442-s001.pdf]

PTPRG and PTPRC modulate nilotinib response in chronic myeloid leukemia cells

SUPPLEMENTARY MATERIALS

Quantitative PCR for PTP gene expression

Primers (Supplementary Table 1) were purchased from Sigma-Aldrich (Deisenhofen, Germany). PCR products were validated for correct size by gel electrophoresis and by DNA sequencing. PCR reactions were carried out in a Roche LightCycler480 system in a 96-well format using 2x Maxima SYBR Green/ROX qPCR Master Mix from Thermo Scientific (#K0223). Primers were used in a final concentration of 0.3 µM. QPCR was carried out with an initial denaturation step of 10 min, 95°C, followed by 40 cycles with 15 sec, 95°C; 30 sec, 55°C; and 30 sec, 72°C. Beta-glucuronidase (*GUSB*) and Beta-2 microglobulin (*B2M*) were used as control genes. Each gene was analyzed in duplicates, relative mRNA expression was calculated as $2^{-\Delta CT}$, using the mean CT value of *GUSB* and *B2M*.

Expression constructs

A human PTPRG-WT expression construct (coding sequence from reference sequence NM_002841.3) was obtained from Vector Builder (Cyagen Biosciences Inc., Santa Clara, CA, USA). The encoded sequence corresponds to the 1445 aa long splice version of PTPRG (Ensembl ENST00000474889.5). A human PTPN6-WT expression construct was originally obtained from Dr. Axel Ullrich (Max-Planck Institute of Biochemistry, Martinsried, Germany). Both coding sequences were subcloned to pMSCV-IRES-mCherry (Addgene # 52114), and mutated to PTPRG-C1060S and PTPN6-C453S respectively using the QuikChange XL Site-Directed Mutagenesis Kit (Agilent Technologies, Waldbronn, Germany; Cat. #200517) according to the instructions of the manufacturer. The PTPRC expression construct was obtained from Dr. Reiner Lammers (University Hospital Tübingen, Germany). It encodes PTPRC isoform 2 (NP_563578.2) [1]. The PTPRC insert was subcloned into a lentiviral expression vector pLV-mCherry/Bsd, containing a mCherry/blasticidin resistance cassette (Vector Builder, Cyagen Biosciences Inc., Santa Clara, CA, USA). Cells transduced with viral particles produced with respective empty vectors were used as controls. All coding sequences and mutations were confirmed by DNA sequencing. Target cells were infected with freshly produced viral particles using a standard protocol, selected with 7.5 µg/µl blasticidin (Life Technologies, R21001) (only PTPRC-vector and corresponding control), and sorted for equally high expression of mCherry. Cell sets with empty vector, expression vector, and mutated vector (if applicable) were generated and processed in parallel.

shRNA mediated knockdown of PTPN6

Stable KCL-22 cell pools with knockdown of PTPN6 were obtained by lentiviral transduction of KCL-22 cells with viral particles made from pLKO.1 (Sigma-Aldrich) constructs harbouring either nontargeting control (Cat # SHC002; insert: CCGGCA ACAAGATGAAGAGCACCAACTCGAGTTGGTGCT CTTCATCTTGTTGTTTTT), or shPTPN6-A (Cat # TRCN0000006885; insert: CCGGCCCAGTTCATTG AAACCACTACTCGAGTAGTGGTTTCAATGAACTG GGTTTTT), or shPTPN6-B (Cat # TRCN0000006886; insert: CCGGGCATGACACAACCGAATACAACTC GAGTTGTATTCGGTTGTGTCATGCTTTTT) and subsequent selection with 2 µg/ml puromycine (Sigma-Aldrich, P8833).

CRISPR/Cas 9 mediated knockout of PTPN6 and PTPRC

Stable PTPN6 and PTPRC knockout cells were generated by infecting the parental cells with viral particles freshly made from lentiCRISPR v2 plasmid (Addgene #52961) containing target specific gRNA (GAAGA ACTTGCACCAGCGTC for PTPN6 clones A and B; TTACCACATGTTGGCTTAGA or TTAATATTAGATG TGCCACC for PTPRC clones A and B respectively). Cells infected with viral particles made from empty lentiCRISPR v2 plasmid were used as controls. Infected cells were selected using 2 µg/ml puromycin and single cell clones were established by limited dilution and analyzed for absence of the target protein by immunoblotting. Two independent knockout clones were selected for further analysis.

Immunoblotting

Cells were washed with ice-cold PBS and lysed on ice in RIPA lysis buffer (1 % NP40, 1 mM EDTA, 50 mM Tris pH 7.4, 150 mM NaCl, 0.25 % sodium deoxycholate) containing PhosSTOP phosphatase inhibitor cocktail (Roche, Mannheim, Germany; Cat. # 04906845001) and cOmplete protease inhibitor cocktail (Roche, Mannheim, Germany; Cat. # 04693132001)

diluted in lysis buffer according to the instructions of the manufacturer. The extracts were sonicated, and the cleared lysates were subjected to SDS-PAGE and transferred to nitrocellulose membranes using tank blot. Membranes were first incubated with the phospho-antibodies as applicable, and subsequently stripped for detection of respective total proteins. Antibodies were obtained from the following companies: Cell Signaling Technology (New England Biolabs GmbH, Heidelberg, Germany): pBCR-ABL1 (pABL #2861), BCR-ABL1 (ABL #2862), pSFK (#6943), pERK 1/2 (#9106), ERK 1/2 (#9107); SANTA CRUZ BIOTHECHNOLOGY (Heidelberg, Germany): PTPN6 (sc-287), PTPRC (sc-25590), p27 (sc-1641), Bcl-2 (sc-7382), cSRC (sc-18), Lyn (sc-7274), STAT5 (sc-835); Sigma-Aldrich: actin (A5441); BIOZOL: vinculin (BZL03106); BD Biosciences (Heidelberg, Germany): pSTAT5 (BD611964). The PTPRG antibody was generated in one of our laboratories as described before [2]. Quantification of blots was done using Fujifilm Multi Gauge software. Intensity of phosphorylation signals was divided by intensity of respective blot for the total proteins. To normalize levels of p27 and Bcl-2, specific signals were divided by the signal for actin. For each individual experiment, the respective signals are reported relative to EV or control cell signals. The mean of these relative phosphorylation signals of $n = 3$ independent experiments is reported +/- standard deviation (SD).

REFERENCES

1. Lammers R, Bossenmaier B, Cool DE, Tonks NK, Schlessinger J, Fischer EH, Ullrich A. Differential activities of protein tyrosine phosphatases in intact cells. J Biol Chem. 1993; 268:22456–22462.
2. Della Peruta M, Martinelli G, Moratti E, Pintani D, Vezzalini M, Mafficini A, Grafone T, Iacobucci I, Soverini S, Murineddu M, Vinante F, Tecchio C, Piras G, et al. Protein tyrosine phosphatase receptor type {gamma} is a functional tumor suppressor gene specifically downregulated in chronic myeloid leukemia. Cancer Res. 2010; 70:8896–8906.

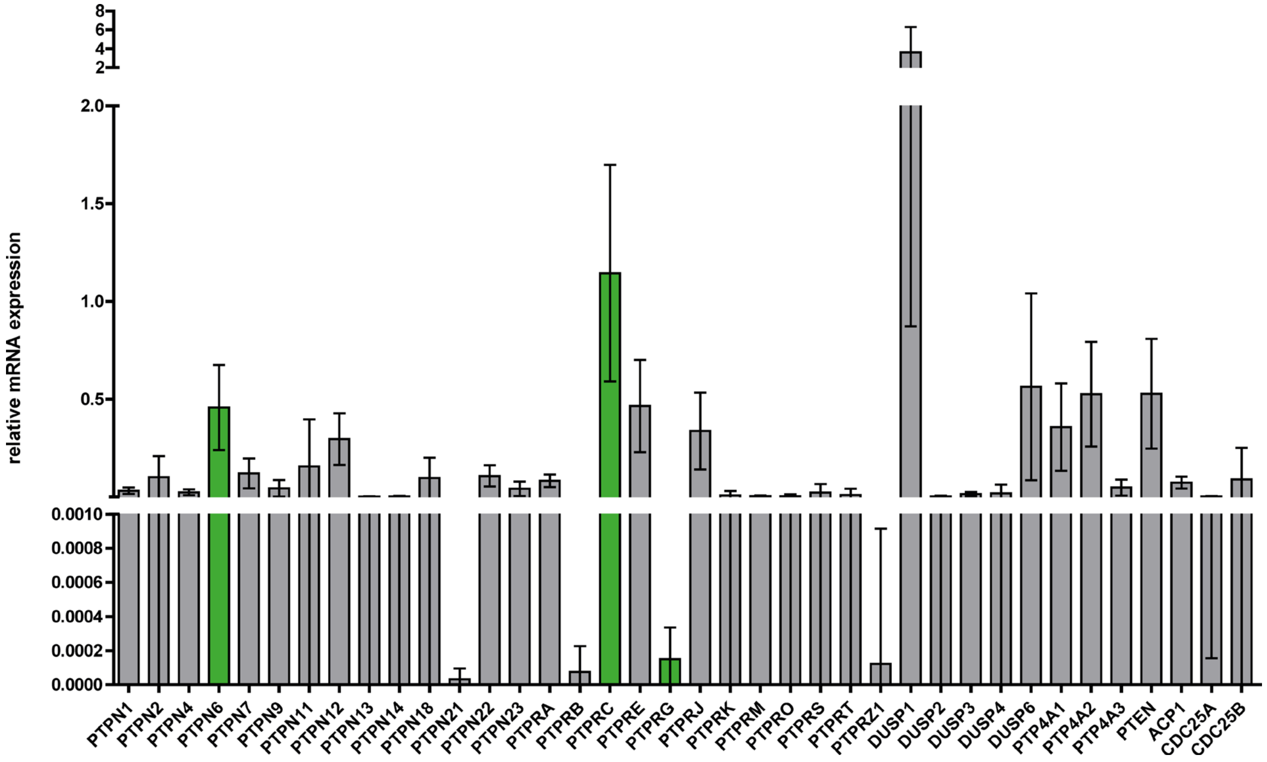

Supplementary Figure 1: PTPs are differentially expressed in CML patients. Total leukocytes of 66 newly diagnosed CML patients were isolated and mRNA levels of 38 PTPs were analyzed using RT-qPCR. *GUSB* and *B2M* were used as control genes. Shown here is the mean relative mRNA level (of n = 66 patients) of all 38 PTPs +/- standard deviation (SD) with technical duplicates for each PTP per patient. The PTPs further analyzed in this manuscript, PTPN6, PTPRC, and PTPRG are highlighted in green.

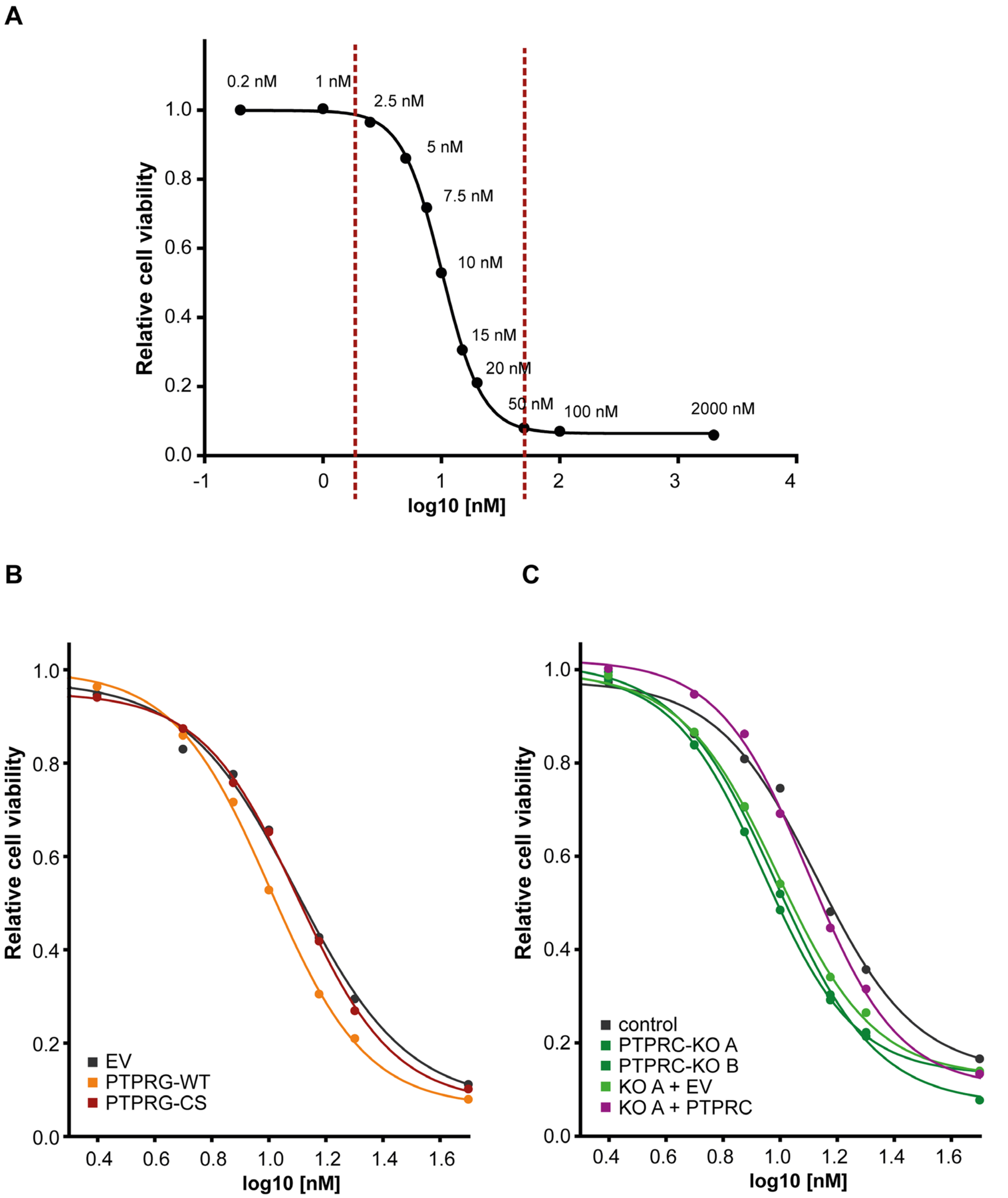

Nilotinib	Experiment A		Experiment B	
	IC_{50}	95% confidence intervals	IC_{50}	95% confidence intervals
EV	12,5	11,49 to 13,59	12,07	11,18 to 13,03
PTPRG-WT	10,09	9,785 to 10,41	9,515	8,614 to 10,51
PTPRG-CS	12,62	11,54 to 13,8	12,13	11,17 to 13,17

Nilotinib	Experiment A		Experiment B	
	IC_{50}	95% confidence intervals	IC_{50}	95% confidence intervals
control	13,39	12,11 to 14,8	12,29	11,08 to 13,62
PTPRC-KO A	9,706	9,25 to 10,18	9,87	8,985 to 10,84
PTPRC-KO B	8,726	8,522 to 8,934	8,51	8,052 to 8,995
KO A + EV	9,791	9,353 to 10,25	9,247	8,618 to 9,921
KO A + PTPRC	12,58	11,92 to 13,28	12,74	11,99 to 13,53

Supplementary Figure 2: Graphical view of dose-response curves and report of IC_{50} 95 % confidence intervals. (**A**) representative fitted dose-response curve obtained for nilotinib. Curve fitting and IC_{50} determination was done using SigmaPlot 13.0. For more clarity, only the dose range shown between the dotted lines is shown in B and C. (**B**) Fitted dose-response curves of one representative experiment with EV, PTPRG-WT, or PTPRG-CS expressing cells. (**C**) Fitted dose-response curves of one representative experiment with control cells, PTPRC-KO A, PTPRC-KO B, PTPRC-KO cells transduced with EV (KO A + EV) or PTPRC-KO cells re-expressing PTPRC (KO A + PTPRC). The tables shown in B and C are reporting the calculated IC_{50} values of two independent experiments and their respective 95 % confidence intervals.

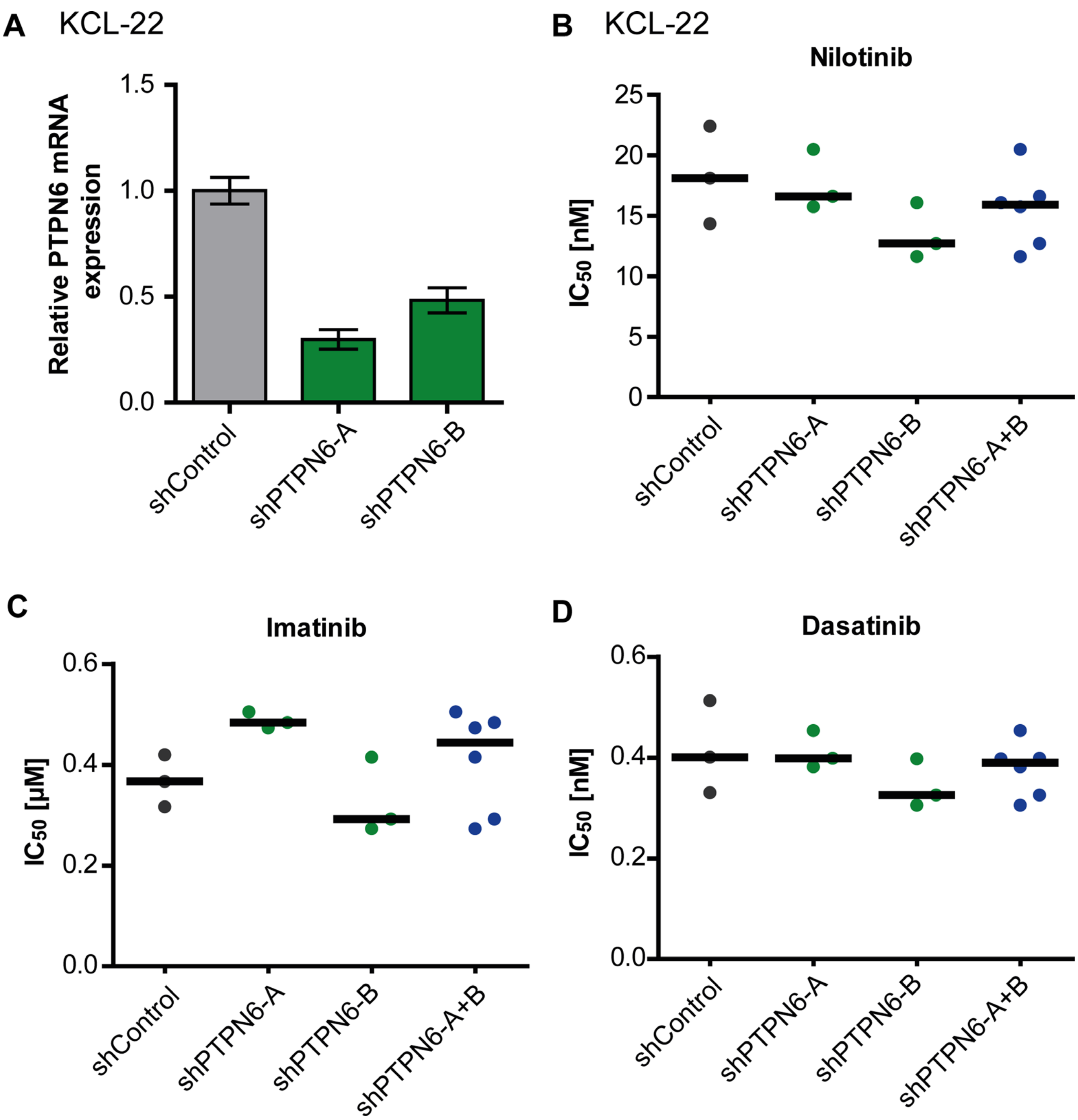

Supplementary Figure 3: ShRNA mediated knockdown of PTPN6 in KCL-22 cells. Cells were lentivirally transduced with either nontargeting control (shControl) or two different shRNA constructs (shPTPN6-A and -B). Knockdown efficiency in the two cell pools was determined by RT-qPCR. Relative PTPN6 mRNA expression is shown +/- standard deviation (SD) of one experiment with duplicate technical replicas (**A**). Cell pools were analyzed for IC_{50} of nilotinib (**B**), imatinib (**C**), or dasatinib (**D**). Each dot represents IC_{50} value of one experiment, black bars show median of n=3 independent experiments (shControl, shPTPN6-A, and shPTPN6-B). ShPTPN6-A+B summarizes the values obtained for the two knockdown cell pools A and B (n = 6).

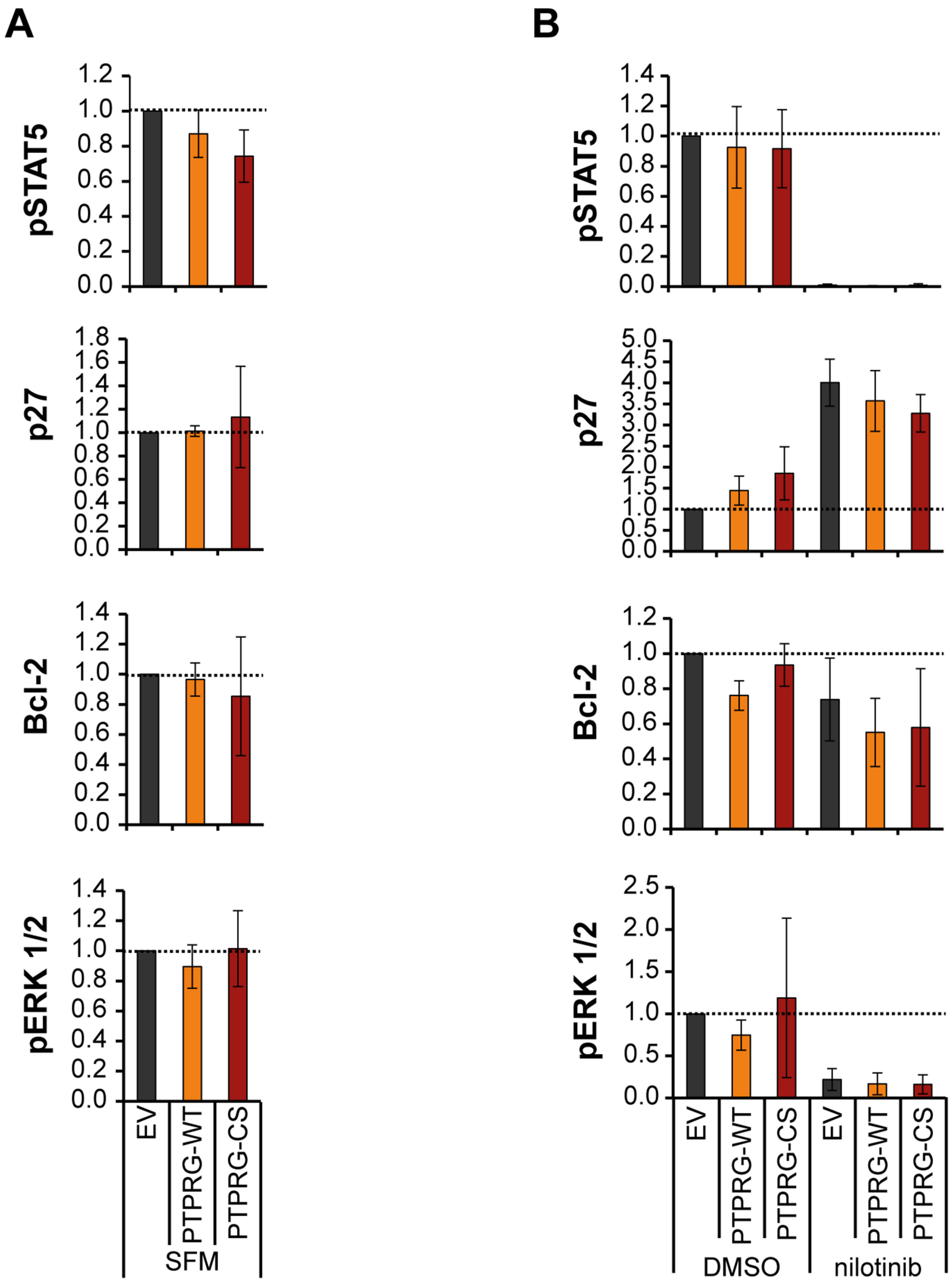

Supplementary Figure 4: Nilotinib treatment and PTPRG overexpression affect BCR-ABL1 signal transduction in the same direction. K562 cells overexpressing PTPRG-WT or PTPRG-CS were starved from serum for 4h (SFM) (**A**), or treated with nilotinib (1 µM, 24 h) or DMSO in presence of 10 % FCS (**B**). Cell lysates were prepared and subjected to SDS-PAGE and immunoblotting. Shown are quantifications of blots from *n* = 3 independent experiments. The phospho-signal was divided by the respective total protein signal (pSTAT5, pERK 1/2) or the signal of total protein was divided by respective actin signals (p27, Bcl-2). The signals of PTPRG-WT and PTPRG-CS are reported relative to the EV cell signal of each experiment. Shown is the mean relative intensity of *n* = 3 experiments +/- standard deviation (SD). Representative blots and quantifications of pBCR-ABL1, pcSRC, and pLyn are shown in Figure 5.

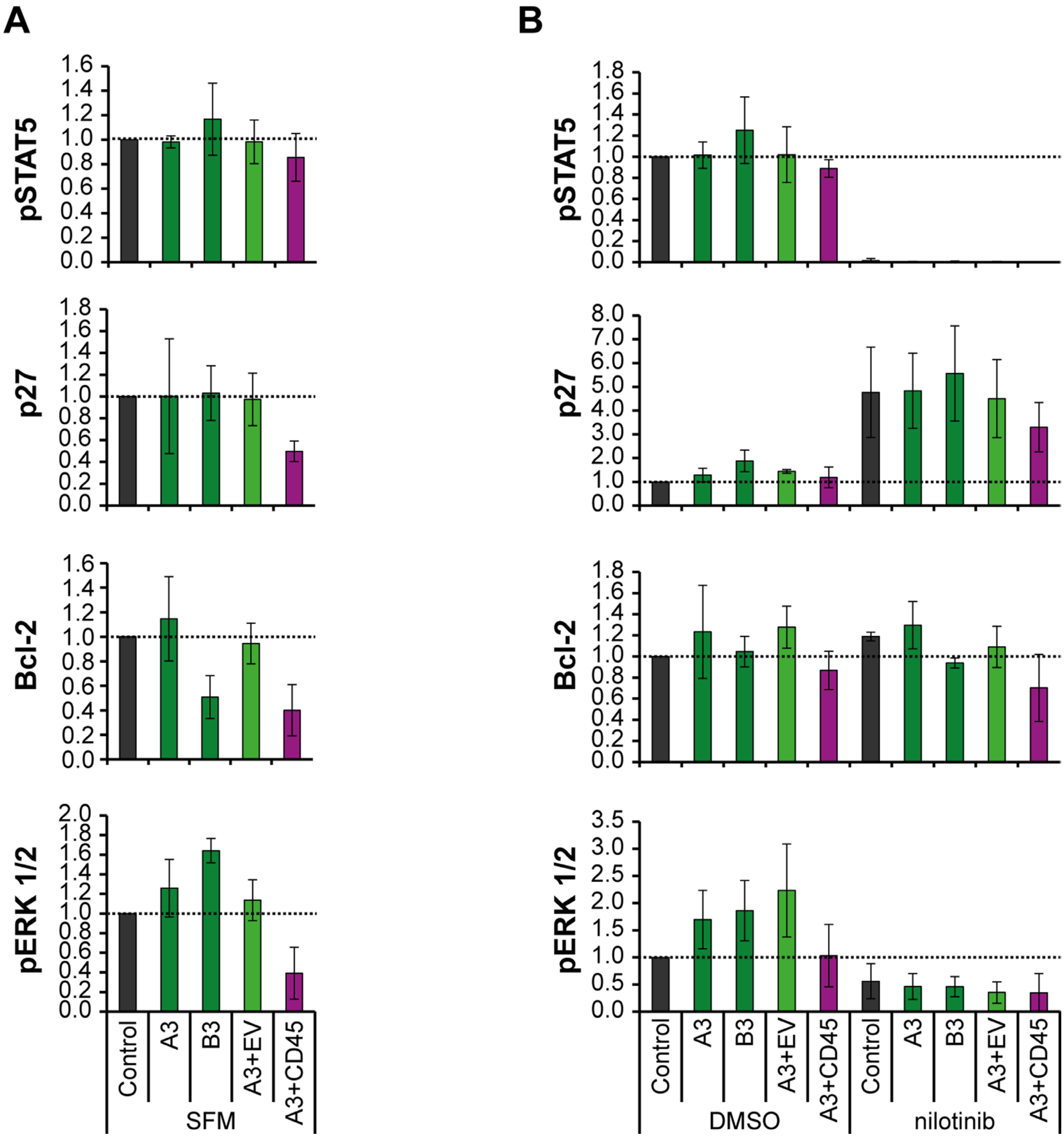

Supplementary Figure 5: Nilotinib treatment and PTPRC deficiency affect aspects of BCR-ABL1 signal transduction in the same direction. K562 cells with PTPRC knockout and respective PTPRC re-expressing cells were starved from serum for 4h (SFM) (**A**), or treated with nilotinib (1 µM, 24 h) or DMSO in presence of 10 % FCS (**B**). Cell lysates were prepared and subjected to SDS-PAGE and immunoblotting. Shown are quantifications of blots from $n = 3$ independent experiments. The phospho-signal was divided by the respective total protein signal (pSTAT5, pERK 1/2) or the signal of total protein was divided by respective actin signals (p27, Bcl-2). The signals of engineered PTPRC KO and re-expressing cells are reported relative to the control cell signal of each experiment. Shown is the mean relative intensity of $n = 3$ experiments +/- standard deviation (SD). Representative blots and quantifications of pBCR-ABL1, pcSRC, and pLyn are shown in Figure 6.

Supplementary Table 1: PTPs and control genes used for RT-qPCR analysis

Gene	Common name	Primer forward	Primer reverse	Size
PTPN1	PTP1B	CGTTAAAATGCGCACAATAC	GCTGTCGCACTGTATAATATG	120 bp
PTPN2	TC-PTP	GGCACCTTCTCTCTGGTAGA	TCTCAGTTGATCTGGGGTCT	129 bp
PTPN4		TACGATCAGTCAGAGAACTTG	GGAGATAAGCCTATGTGTTG	119 bp
PTPN6	SHP-1	CAAGAACATTCTCCCCTTTG	TCTTGATGTAGTTGGCATTG	98 bp
PTPN7		AGGAGAAATGTGTCCACTAC	AAAGAGGATGTGCTTTACTG	155 bp
PTPN9		AGAAACTCGAAGGAAGGAAG	GATGGTGAATTTTCCACTGAG	88 bp
PTPN11		CCTGAATTTGAAACCAAGTG	ACATTTACTCTTTCCTCTCTCC	168 bp
PTPN12		ATGAATCTCGTAGGCTGTATC	CCTCATTAAGCTTATCATGTCC	101 bp
PTPN13	FAP1, PTP-BAS	GAAGAACCAGTTCGAAGATAC	ACTTAAGGTTTCATCTACCCC	171 bp
PTPN14		CAGAATTGGAAGACAAGCGG	TAAAACTTGTGTCGTGTGGC	177 bp
PTPN18		AGGAGAAGTGGCTGAATGAG	GTAGCTGGTACACAGAACGG	84 bp
PTPN21		ATTTAGGTGGCATGACATTG	CAACACAGAGTCTCCAAATG	137 bp
PTPN22	Lyp	GTGAAACTCGAACTATCTACC	TCTTGGTAACAACGTACATC	112 bp
PTPN23		CTTTGAGGGCTGTAGTGTC	CGCTCCAAGGTTGTAGAGAA	187 bp
PTPRA	RPTPα	CAACAATGCTACCACAGT	AAGAGAAGTTAGTGAAGAAGTT	121 bp
PTPRB		CTCTGTGGACATTTATGGAG	ACTGGATCTCTGTGATACTC	195 bp
PTPRC	CD45	CATATGACTATAACAGAGTGCC	ATGTATTTGCTTGGTTCCTC	118 bp
PTPRE		ATGACCATTCTAGGGTGATTC	CCATCTATGTAGGAAGCATTG	79 bp
PTPRG	RPTPγ	GACGAGAAGGAGAAGACGTT	AACAGGTAAAGGCTATCGGG	89 bp
PTPRJ		ATAGGCAATGAGACTTGGGG	CATGGCTTCCAATGCTTTCA	172 bp
PTPRK		CTTGAAACTGATACTTCAGGTG	TTAGGCAGTGGATAATCGTC	80 bp
PTPRM	RPTPμ	GGCCAGATGACACAGAGATA	CATGCACACCTCTCTTTTCAA	111 bp
PTPRO		ATTGAAAATCTGGTTCCTGG	ACAATAGCAAATGTCACAGG	101 bp
PTPRS		CAGAGGATGAGTACCAGTTC	CTTTAGGTTGCATAGTGGTC	76 bp
PTPRT		AGAGGAGCAATATGTGTTTG	GATATTGTAGTAGAGAGAACGG	103 bp
PTPRZ1		TGAGGTGCTGGACAGTCATA	GCTCAGGAGCTGGAATTGTT	100 bp
DUSP1		ACTACCAGTACAAGAGCATC	GATTAGTCCTCATAAGGTAAGC	183 bp
DUSP2		CAACTTCAGTTTCATGGGG	CTGAAACTCTGAGGAGGTAG	184 bp
DUSP3		TTGGCTCAAAAGAATGGC	ATCATGAGGTAGGCGATAAC	86 bp
DUSP4		TATCAGTACAAGTGCATCCC	ATCGATGTACTCTATGGCTTC	84 bp
DUSP6		TGGTGTCTTGGTACATTGCT	CGTTCATCGACAGATTGAGC	92 bp
PTP4A1		ACGAAGATGCAGTACAATTC	TCAAGTTCCACTTCCAGTAG	183 bp
PTP4A2		TACGTTGCACATTTATGGCG	GCTCACTTGTCAGCGAAAAT	82 bp
PTP4A3		AGCACCTTCATTGAGGAC	GCGTTTTGTCATAGGTCAC	79 bp
PTEN		GGCTAAGTGAAGATGACAATC	GTTACTCCCTTTTTGTCTCTG	169 bp
ACP1		CTGTTTGTGTGTCTGGGTAAC	TGATCGGTTACAAGTTTCCTG	74 bp
CDC25A		AGAAGAATACATTCCCTACCTC	CAAGAGAATCAGAATGGCTC	76 bp
CDC25B		ATTGTAGACTGCAGATACCC	GAAAATGAGGATGACTCTCTTG	147 bp
GUSB		TAGAGCAGTACCATCTGGGT	GCTGCACTTTTTGGTTGTCT	160 bp
B2M		TACACTGAATTCACCCCCAC	GATGCTGCTTACATGTCTCG	110 bp
